# Facing Disruptive Changes With Informal Workplace Learning Strategies: The Experience of European Companies

**DOI:** 10.3389/fpsyg.2022.889850

**Published:** 2022-05-26

**Authors:** Francesca Amenduni, Essi Ryymin, Katja Maetoloa, Alberto Cattaneo

**Affiliations:** ^1^Department of Educational Technologies in Vocational Education and Training, Lugano, Switzerland; ^2^HAMK Edu Research Unit, Hämeenlinna, Finland

**Keywords:** disruption, informal learning, digital technologies, workplace, mixed method analysis

## Abstract

Industries are currently experiencing several kinds of disruptive changes, including digital transformation and environmental and health emergencies. Despite intense discussion about disruptive changes in companies, the impact of such changes on workplace learning is still underexplored. In this study, we investigated the impact of disruptive changes on informal learning practices according to the perspectives of employers, employees and adult educators. Informal learning was operationalised along a continuum between organised informal learning (led by an instructor and intentional) and everyday informal learning (led by contextual factors, accidental, and unintentional). Fifty-five companies’ representatives (average age = 43.2 years; SD = 11) from three European countries (Finland, Switzerland, and Italy) and four industrial fields (bioeconomy, tourism, textile and building sectors) were interviewed. The interviews were further triangulated with questionnaires collected by employees from the same companies (*N* = 141; average age = 40.2 years, SD = 17.8). Questionnaire data were used to collect detailed information on individual informal workplace learning (IWL) strategies and digital technologies adopted in organised informal learning. The interview data were analysed using qualitative content analysis. A coding scheme was developed with five macro-categories organised into 23 sub-categories. Occurrence and co-occurrence analysis were performed to identify which individual and organisational factors and approaches support most learning, according to interviewees. Interviewees reported the possibility of interacting with colleagues and being autonomous as the main sources of everyday informal learning processes. Employees from the same companies reported model learning, vicarious feedback, and applying someone’s own ideas as the most frequent IWL strategies. Organised informal learning was mainly based on knowledge transfer, which reflects passive cognitive engagement by employees. Specifically, digital technologies in organised informal learning were poorly used for supporting reflection, constructive processes, and collaborative knowledge construction. The results suggest that participants believed that higher forms of cognitive engagement are possible only within face-to-face organised informal training or in everyday informal learning. Possible explanations of the results and practical implications are discussed.

## Introduction

Industries are currently experiencing, to a different extent, *disruption*, which can be defined as the action of completely changing the traditional way an industry or market operates by using new methods or technology (Kilkki et al., 2018). These actions are taken in response to a complex set of interrelated factors, including technological advancement (Arntz et al., 2016), environmental challenges and health emergencies (Rapaccini et al., 2020). Embracing disruption can be worthwhile because it has the potential to improve the quality of employers’ condition and to realise more sustainable and efficient business processes. However, in some cases, disruption may speed the transition towards decline. Examples include industries that fail to recognise the potential of disruptive innovations (Moreau, 2013) or companies that introduce potentially disruptive technological-based innovation without changing their organisational cultures (Sabatier et al., 2012). Indeed, dealing with disruption means being prepared to face it properly, considering not only tangible elements (e.g. new technological systems and infrastructures) but also intangible elements, such as professional practices and skills (Phaal et al., 2011). Therefore, workplace learning processes are a critical component of effective management of disruptive changes. Learning in the workplace is a complex phenomenon involving the interaction between individual and organisational levels. Much research on workplace learning has focused on the informal components of workplace learning (Tynjälä, 2008; Illeris, 2011; Clark et al., 2018; Decius et al., 2019). There are several ways to define informal workplace learning. The most common way to define informal learning is by contrasting it with the concept of formal learning, which is education provided by a recognised institution acknowledging a formal certificate and organised by an instructor. However, more recently, researchers have found that a clear distinction between formal and informal learning has not been grounded in the actual reality of people’s learning. For this reason, Colley et al. (2003) developed the notion of a continuum of learning formality. The continuum presents four main attributes that define the degree of formality of a specific learning situation: *the driver of the learning process* (driven by a teacher, a learner, or by contextual factors), the degree of awareness and *intentionality* of the people involved in the learning process, the *content* to acquire (from learning outcomes defined through a curriculum to content based on individual needs or social norms and practices), and the *place* where learning happens (school, company, home and communities). By combining the four factors, it is possible to identify two opposite ends of the workplace learning informal continuum: on one end is *everyday informal learning*, which is driven by contextual factors, not planned *a priori*, and with a low level of intentionality. On the other end is *organised informal learning*, which is intentional and strategically supported by the companies, providing a structure for the everyday workplace experience, for example, by supporting reflective observation of professional experience (Kolb, 1984; Billett, 2011).

Informal learning in the workplace has been extensively studied in the last 20 years. However, there is poor knowledge about the impact that disruption could have on the different configurations of informal workplace learning, from everyday informal learning to organised informal learning (Fischer et al., 2018; Harteis, 2018). To fill this gap, the goal of this research was to explore the impact of disruptions on informal learning at work in European industries. Specifically, we were interested in exploring two research questions:

1. Which factors support everyday informal learning according to employers, employees, and adult educators employed in companies that deal with disruption?
2. Which approaches do companies that deal with disruption adopt in organised informal learning? How do they integrate digital technologies into organised informal learning?

In the theoretical part of this paper, we present the inter-relation between the individual and the organisational factors that can support informal learning at work and the role that digital technology could play in bridging different forms of informal learning. We then present the results of an interview study aimed at exploring informal learning strategies activated in response to disruptive changes in companies.

### Individual Factors in Informal Workplace Learning

Any learning process involves people who learn and their personal characteristics. Illeris (2011) identified two main individual factors that play a role in informal workplace learning. The first are incentives, or the reasons why people learn. Incentives are necessary because learning, like any other kind of mental process, requires the mobilisation of energies (Fernet et al., 2008). The second factor is the strategies individuals use to acquire content. In contrast with what happens in formalised learning contexts, such as schools, in the workplace learning contents are not always elicited by trainers or supervisors, but they are acquired by the employees in their everyday activities through self-driven informal learning strategies, such as co-operating and interacting with colleagues, working with clients or tackling challenges and new tasks (Billett et al., 2005). Decius et al. (2019) attempted to systematise the different kinds of individual informal workplace learning (IWL) strategies that employees adopted in their everyday informal learning based on the Dynamic Model of Informal Learning by Tannenbaum et al. (2010). The model of Decius et al. (2019) contains eight factors of informal learning: six strategies and two kinds of motivations for learning. The six IWL strategies are *trying & applying one’s own ideas, model learning, direct feedback, vicarious feedback, anticipatory reflection,* and *subsequent reflection*. *Trying & applying one’s own ideas* refers to learning by doing the job. *Model learning* is based on learning from others by observing their behaviour and adapting one’s own behaviour to those observations. Feedback is divided into *direct feedback,* which refers to asking a colleague to directly assess one’s performance, and *vicarious feedback,* which refers to self-assessment of one’s performance by asking a colleague to describe a similar professional experience. *Anticipatory reflection* occurs before the task, for example, when anticipating new hindrances during the execution of a task to adapt to changed working conditions, and *subsequent reflection* is the personal considerations or thoughts after finishing the task.

Decius et al. (2019) described these six IWL strategies in the context of *everyday informal learning*, characterised by a low level of intentionality and driven by contextual and accidental factors. However, it is also possible to classify the individual strategies and behaviours that people adopt in the context of *organised informal learning*, which means that it is deliberatively facilitated by a trainer or a supervisor within the company. In this paper, we use the ICAP model (Interactive, Constructive, Active and Passive) to define the different individual strategies and behaviours people adopt in the context of organised informal learning. The ICAP model has been developed and tested in formal learning contexts, such as schools, to classify different forms of cognitive engagement that can be elicited by a teacher. This model can be particularly useful for describing the companies’ approaches when they deliberately provide training. According to the ICAP framework, it is possible to distinguish four different types of learning activities based on the observation of learners’ behaviours: interactive, constructive, active and passive. Each of these activities reflects different levels of learners’ cognitive engagement that is defined as the investment of cognitive effort in the learning process (Chi and Wylie, 2014; Chi et al., 2018). In *passive* learning activities, learners work with knowledge in a merely receptive manner (e.g. employees watch an instructional video without interacting with or manipulating it). *Active* learning occurs when learners have hands-on opportunities to interact with and practice given instructional material and content (e.g., interacting with a quiz in a video or manipulating objects in a virtual reality environment). In *constructive* learning, learners individually create new knowledge and new links between elements of knowledge (e.g. creating project works, prototypes and solutions). *Interactive* learning occurs when learners collaborate with others with the purpose of building new knowledge (e.g. sharing ideas, discussing their argumentations, constructing a joint point of view). The ICAP framework suggests that cognitive learning processes become increasingly sophisticated, moving from passive to interactive learning activities (Chi et al., 2018). Besides the four learning modalities described by ICAP, in this study, we also considered *reflective learning*, which is a specific modality of work-based learning in which the learner is invited to reflect upon their concrete experience (Schon, 1983), for example, by commenting on the video recording of their own professional activity (Tripp and Rich, 2012).

### Organisational Factors in Informal Workplace Learning

Workplace learning is not only dependent on individual factors but also on the learning opportunities provided by the social and cultural context in which learning occurs. In this direction, the workplace learning environment is constituted by two elements: production and community (Illeris, 2011; Tynjälä, 2013).

The production element refers to the organisation of work, such as the division of labour and workload, which affects the possibility for employees to make decisions and to be autonomous (Kirby et al., 2003). The organisation of work is a very important contextual factor for workplace learning. For instance, in the traditional Fordist organisation, the workers have narrow job descriptions, repetitive tasks, controlled procedures and few opportunities for autonomous decision-making. At the other end of the continuum, there are organisations in which the job continuously provides new challenges and learning opportunities. In these workplaces, workers are rotated among roles, tasks are carried out by collaborative and self-managed teams with a lot of autonomy, and workers are encouraged to share their expertise and develop their work. The organisation of work could be related to an organisational learning culture and orientation towards learning, which therefore creates a space to exert different kinds of IWL strategies (Fuller and Unwin, 2011).

The community element refers to a group of interdependent employees who share common tasks and professional objectives and values and include opportunities for social interactions with supervisors, managers (Kirby et al., 2003) and colleagues (Billett, 2001). Illeris (2011) acknowledged that work-related learning takes place not only within the company but also through external social networks created by the employee and the company by interacting with external clients and/or suppliers (Knight, 2002) and through informal networks. Connectivity with external people and organisations is an important source of transformative learning (Engeström, 2001).

The mutual relationship between organisational factors, such as the organisation of work, and individual learning strategies is included in different theoretical models (Fuller and Unwin, 2011; Illeris, 2011; Tynjälä, 2013) but poorly explored in an empirical perspective (Panadero, 2017) or explored only through small-scale case studies (Barabasch and Keller, 2020). One of the rare examples of larger-scale empirical research is that of Froehlich et al. (2015), who found that the organisational learning culture moderates the effects of individual learning approaches—categorised as deep, surface-rational, and surface-disorganised learning (Kirby et al., 2003)—on learning outcome. The authors’ focus, however, was on the individual learning approach rather than on the IWL strategies, which are more related, by definition, to the workplace context (Decius et al., 2019). Moreover, Froehlich et al. (2015) did not study the mutual relationship between organisational factors and individual learning strategies. In this respect, Nurmala (2014) found that when employees perceive their organisational culture as oriented towards learning, they are also more engaged in informal learning activities. More recently, Kittel et al. (2021) found that organisational factors, such as organisational learning culture, job characteristics autonomy and feedback, can empower employees to apply self-regulated strategies, which can be defined as a specific form of everyday informal learning (Colley et al., 2003). Thus, although there is some empirical evidence that organisational factors, such as learning culture, might be related to informal individual learning strategies, more research is needed to examine the mutual relationships between organisational and IWL strategies, which is one of the aims of the current study.

### Technology as a Bridge Between Formal and Informal Workplace Learning

The many opportunities for learning informally in the workplace have been increased by the ubiquitous nature of digital technology (Brown and Mbati, 2015) by making the boundaries between informal and formal learning even more blurred and crossable (Littlejohn and Margaryan, 2014; Egloffstein and Ifenthaler, 2017; Ang et al., 2018; Clark et al., 2018). Emerging opportunities for digital learning include game-based learning, simulations, massive/corporate open online courses (MOOC, cMOOC), social networks, learning analytics, and mobile and augmented applications (Ifenthaler, 2018). According to Wong and Looi (2011), mobile devices create ‘seamless learning spaces’, providing continuity to the learning experience across learning venues and between individual and social learning. The widespread adoption of technology in many facets of modern life has created an increasing urgency to recognise the importance of adult informal learning, especially when it happens in a digital environment (Ang et al., 2018). However, there is poor empirical evidence concerning the strategies, more or less deliberate and structured, that people adopt when they learn in the workplace by using digital technologies. Ang et al. (2018) investigated learning behaviours associated with the use of technologies in a dual programme in which learners are working full-time and studying part-time for a tertiary degree. They found similar patterns between school and workplace use of digital technologies, mainly oriented to ‘receiving’ information such as accessing newsfeeds, listening to podcasts, and downloading content from the internet (which corresponds to the passive mode of the ICAP framework). More research is required to understand learners’ strategies in the adoption of digital technologies for informal workplace learning. In the context of this paper, we use the ICAP framework to categorise how companies adopt digital technologies in informal workplace learning when faced with disruptions.

## Materials and Methods

### Research Context: Disruption in European Companies

The starting point of this research project was to choose industries from a variety of sectors that are facing disruptions and to provide a representation of different European areas (North, Central and South Europe). To identify companies to include in our research, we followed the policy papers developed by the European Commission on disruption in European companies. We then looked for companies based on the representativeness of the industrial sectors in each partner’s region (Lombardy in Italy, Ticino in Switzerland and Kanta-Häme in Finland), eventually selecting companies based on their availability to participate in the research. We managed to include four industrial sectors from the three countries: textile (Italy and Switzerland), tourism (Italy and Switzerland), building sector (Switzerland) and bio-economy (Finland). In this section, we describe the reasons why these four sectors are considered to be facing disruption.

The bioeconomy covers multiple scientific fields and interrelated perspectives, highlighting biotechnology, bio-resources and bio-ecology (Bugge et al., 2016). Several global policy papers (European Commission, 2012; Klitkou et al., 2017; OECD, 2020a) have reflected on how the bioeconomy can meet digitalisation as a disruptive process. Digitalisation in the bioeconomy is connected to applications of digital technologies, digitised data and changing business models. This is happening alongside a radical change in consumer behaviour, for example, with the emergence of a circular economy (Klitkou et al., 2017), which is important because it aims at eliminating waste and the continual use of resources by employing reuse, sharing, repair and recycling (Ryymin, 2021).

The tourism sector is influenced by several megatrends, including demographic developments, technological innovations (impacting business models, needed competences and tourist experiences) and pressing demand for more sustainable practices in the consumption, production and development of tourism (Next Tourism Generation Alliance, 2019). The emergence of digital platforms for peer-to-peer accommodation rentals is an example of a disruptive innovation that has affected the tourist sector increasingly over the last 20 years (Zach et al., 2020). Further, tourism is also one of the sectors hardest hit by the COVID-19 pandemic (OECD, 2020b).

The construction sector is facing demographic changes as well as a shortage of skilled labour force. According to the Cedefop (2016), about one million new and replacement workers will be needed by 2025. Additionally, the skills needed in construction are likely to change to meet the demands for ‘green’ and energy-efficient buildings. Important trends for the construction sector includes digital innovations in construction (e.g., 3D printing and drones), the use of building information modelling (BIM), and the transition towards a circular economy.

The textile sector has also experienced a series of major transformations in recent decades. The European Commission recognises that the textile ecosystem encompasses several interlinked activities that produce a wide variety of final products. The industries that compose this ecosystem are important pillars of the EU economy, and therefore, the textile ecosystem is one of the industrial ecosystems that the Commission has identified as strategic in the recovery from the COVID-19 pandemic and for building a stronger single market. In a recent study focusing on the textile sector, key findings highlight the interlinked and international composition of the textile sector, its significant contribution to environmental sustainability and circular economy, the rapid growth of e-commerce in recent years, and the effects of the COVID-19 pandemic on both the textile ecosystem and the fashion industry (European Commission, 2021).

### Participants

To understand the complex phenomenon connected to informal workplace learning, three target groups were included from each company: employees, employers and adult educators. The perspective of each group was needed to collect the whole picture of informal workplace learning processes that encompass individual and organisational dynamics, as well as organised learning programmes and more spontaneous learning.

Table 1 provides a description of the 55 participants interviewed (CH = 24, ITA = 15, FI = 16, average age = 43.2 years, SD = 11, female = 25) and the 141 people who completed the questionnaire (CH = 14, ITA = 54, FI = 73, average age = 40.2 years, SD = 17.8, female = 71).

**Table 1:** Participants in the interview (INT) and the questionnaire (QUEST) study.

	Finland		Switzerland		Italy	
	INT	QUEST	INT	QUEST	INT	QUEST
**Bio-economy**						
Employee	9	38				
Employer	4	7				
Adult Trainer	3	28				
**Textile**						
Employee			2	–	–	10
Employer			4		3	2
Adult Trainer			–	–	2	1
**Tourism**						
Employee			3	1	7	22
Employer			5	3	2	10
Adult Trainer			5	2	1	9
**Building sector**						
Employee			3	1		
Employer			1	3		
Adult Trainer			1	4		
Total	16	73	24	14	15	54

### Data Collection

Data were collected between March and June 2021. A multi-method approach was adopted: interviews with adult educators, employees and employers were triangulated with quantitative data collected through a questionnaire.

Employees, employers and adult trainers were interviewed following an interview guide with specular questions for the three roles. The interview questions were built following the theoretical framework provided by Vermersch (2019), which is aimed at supporting the interviewee’s awareness through a detailed description of personal experiences, actions, and practices. At the beginning of the interviews, participants were invited to reflect upon the disruptions they had faced in their work in the past 5 years, identifying concrete examples and experiences. They were then asked to tell us: (1) how they faced these challenges in everyday informal learning; (2) how they were engaged by trainers in organised informal learning programmes—and to what extent digital technologies are adopted for this purpose; and (3) which individual and organisational factors supported them in acquiring new competences. At the end of the interview, the participants were asked to reflect upon and anticipate possible future disruptions and the impact of these disruptions on their jobs. The interviews lasted an average of 38.7 min (SD = 13.9).

A questionnaire was submitted to the same companies that agreed to take part in the interviews. The questionnaire was composed of 34 questions that included basic information and scales related to individual and organisational factors that can affect learning at work: occupational self-efficacy (Rigotti et al., 2008), approaches to learning at work (Kirby et al., 2003), IWL strategy (Decius et al., 2019), motivation to attend training (Fernet et al., 2008), acceptance of professional training enhanced by web-based tools (Cheng et al., 2011), perception of qualitative job insecurity (Van Hootegem and De Witte, 2019), workplace climate (Kirby et al., 2003), formal training opportunity provided at work, digital technologies adopted in training activities (Chi et al., 2018; CIDP, 2020) and non-formal learning activities (EUROSTAT, 2020). The original English version was translated into three different languages and submitted through Qualtrics software, Version [March 2022] of Qualtrics (2022).

The interview data are considered the main data resources for answering the two research questions reported above. Questionnaire data were used to triangulate qualitative data by collecting more detailed information about IWL strategies (Decius et al., 2019) and digital technologies adopted in organised informal learning (Chi et al., 2018; CIDP, 2020). The IWL scale is composed of 18 items, three items per each IWL strategy: Trying & applying own ideas (TAI), model learning (ML), direct feedback (DF), vicarious feedback (VF), anticipatory reflection (AR) and subsequent reflection (SR). Table 2 includes examples of items for each dimension. For the full scale, refer to Decius et al. (2019).

**Table 2:** Data collected from interviews and questionnaire, respectively, with examples of questions and items.

Topic	Questions of the interviews—examples	Questionnaire items – illustrative examples
Past and future disruptions	What were the biggest transformation/s that you have experienced in your work in the last 5 years?	–
IWL strategies and factors that support learning	What competence did you need to acquire to face these challenges? How did you do it? Can you tell us an episode when you feel that you have learned something at work?	Please indicate the extent to which the following statements describe your behaviour at work (1 = totally disagree, 2 = rather disagree, 3 = rather agree, 4 = totally agree). *I try a different method to solve new tasks at work.* (TAI) *I look at how others work in the company to improve my work.* (ML) *I ask my foreman or head how well I have worked.* (DF) *I ask my colleagues about their experiences at work.* (VF) *Before starting a new task, I think about how to do my work best.* (AR) *When I have finished a new task, I think about how well I have worked.* (SR)
Organised informal learning	Could you please give some examples on learning opportunities provided by your company	Which kinds of digital technologies were included in the courses you attended? You can select more than one option.

### Data Analysis

Qualitative data collected through interviews were analysed following the method of qualitative content analysis (Mayring, 2004). Each interview was audio-recorded and transcribed verbatim. The text of the interviews was chunked into a set of units of meaning, defined as ‘an idea, argument chain or discussion topic’ (Strijbos et al., 2006, p. 31). A total of 1,179 units of meaning were identified in the 55 interviews. Table 3 describes the distribution of the units of meaning across sectors, nations and roles. The researchers developed a coding scheme to code the units of meaning. The coding scheme was developed and discussed iteratively, based on theories of informal workplace learning and directly from structuring content analysis (Miles and Huberman, 1994).

**Table 3:** Distribution of units of meaning across sectors, nations and roles.

	Absolute frequency	%
**Sector**		
Tourism	414	35.1
Textile	237	20.1
Bio-economy	352	29.9
Building sector	176	14.9
**Role**		
Employer/HR manager	365	31.0
Adult educator	254	21.5
Employee	560	47.5
**Country**		
Finland	352	29.9
Italy	136	11.5
Switzerland	691	58.6

The characteristic of the transversal category is that their sub-categories are always coded in association with at least one sub-category of one of the four content macro-categories. Each unit of meaning was coded through a non-mutually exclusive approach. This means that a unit could be coded with as many categories as appropriate. We performed an occurrence (O) analysis of the sub-categories retrieved from the interviews. We applied the Chi square test to see whether there is a significant association between the sub-categories support/inhibit learning and personal/organisational factor and learning approach (Gries and Durrant, 2020), and we used the Cramer’s V to measure the strength of the relationship between the sub-categories (Prematunga, 2012). Cramer’s V is a measure of the strength of association between two nominal variables, which ranges from 0 to 1, and is usually reported in association with a Chi square test (McHugh, 2013). We reported relevant extracts to deepen the occurrence and co-occurrence analysis.

To enhance the validity and reliability of the qualitative analysis, researchers in the different countries arranged two online meetings. During the first meeting, the partners compared their coding on a small sample of data, discussed divergences and found agreement on the interpretation of the coding categories. The second meeting was devoted to inserting emerging coding categories into the coding scheme and again comparing the interpretation of coding. At the end of the analysis cycles, we obtained a coding scheme organised into four content macro-categories and one transversal macro-category for a total of 23 sub-categories (see Table 4).

**Table 4:** Coding scheme for the qualitative content analysis.

Macro-category	Sub-categories	Definition	Example
**Disruption**	Customers/Suppliers needs	Driven by customer behaviour and customers’ new needs	*Customers have new expectations concerning the menu, they expect more vegetarian options*
	Sustainable development	Demands for improvements in environmental quality and energy use	*Our goal for the future is that 80% of the jeans we produce will be made with re-cycled materials*
	Automation	Traditional human-tasks are executed by machines	*Many hotels have already self-check-in solutions and in these cases, receptionists are not anymore required*
	Digital transformation	A fundamental change process, enabled by the innovative use of digital technologies	*A first disruption for us was related with the e-commerce*
	Internet of things	Bridging of the physical and digital world through cyber-physical systems	*We are working more and more on the development of the supply chain involving physical shops and e-commerce shops*
**Personal factors which impact on learning and up-skilling**	Occupational self-efficacy (Rigotti et al., 2008)	The competence that a person feels concerning the ability to successfully fulfil the tasks involved in his or her job	*My new management role required me relational competences that I perceived to not have*
	Personal learning approach (Kirby et al., 2003)	A set of motives and strategies used to achieve desired learning outcomes	*I need to be updated constantly since in law there are always new regulation, especially if we consider the European context*
	Informal workplace strategies (Decius et al., 2019)	Self-directed actions which reflect at least some intent for development, growth, learning, or improvement	*I had never done a video-call before the pandemic. I asked support to my more “digital” colleagues*
	Perception of job insecurity (Van Hootegem and De Witte, 2019)	Perceived threats of subjectively important aspects of the job	*Now the rhythm of the change is so high that if you do not learn, you do not only stop your career growth, but you cannot survive anymore*
	Motivational factors (Fernet et al., 2008)	The intent to develop and improve oneself in the workplace, by acquiring new work-related knowledge	*I decided to follow a post-graduate course because I cannot work as process expert if I do not know companies’ processes are changing*
	Attitude toward technology-enhanced workplace learning (Cheng et al., 2011)	Perceived usefulness of digital technologies for individual and peer learning and intention to use digital technologies for learning and up-skilling.	*At a certain point, people who started working in 2000 had to change their mindset toward technologies. People who did not are less reactive and flexible*
**Organisational factors which impact on workplace learning and up-skilling**	Possibilities for social interaction (Kirby et al., 2003)	The organisational availability to provide guidance and mentoring from supervisors and peers	*I am a person who observe a learn, and I learn by looking at what my colleagues are doing*
	Workload (Kirby et al., 2003)	Perception of heavy workload	*Studying and learning while working is really demanding*
	Promotion of autonomy (Kirby et al., 2003)	Employees have some control in decision making, over what work to do and how to do it.	*My work allows me a certain amount of autonomy and this allows me to use my creativity*
	Connectivity (Engeström, 2001)	Cooperation with external communities (educational, customers, other companies)	*During the last months, I participated in webinars where I met people who work in my same role in different companies from which I learned a lot*
	Rewards	Economic reward, career opportunities	*The companies always provided me the budget to attend continuing training*
**Organised informal learning approach**	Passive (Chi and Wylie, 2014)	Paying attention without overtly doing anything	*We attended webinars in which someone showed us new materials and products*
	Active (Chi and Wylie, 2014)	Manipulating knowledge and learning materials and interacting through contents	*In order to make the right questions, I need to test and touch the materials and products*
	Reflective (Schon, 1983)	Actions are followed or anticipated by reflections	*When I receive negative feed-back I want to understand why. So, I look at what I did wrong, what can I change, and I re-elaborate a bit on my work*
	Constructive (Chi and Wylie, 2014)	Learners individually generate or produce additional externalised outputs or products	*My manager asked me to map the sales order in a flow chart. At first, I thought it was a job of maybe 4–5 h. Actually, it took me almost a month to complete it, because it was more complex than expected. That’s when I realised, I learned something new*
	Interactive (Chi and Wylie, 2014)	Co-creation of knowledge products during workshop through teamwork and group collaboration	*We created an internal file where, we marked everything that we could have done better and then we set areas of improvement*
**Transversal categories**	Inhibit learning—upskilling	The personal/organisational factor is mentioned as something which inhibit learning and up-skilling	*Sometimes more expert people are jealous of their knowledge and you do not learn anything from them*
	Support learning—upskilling	The personal/organisational factor is mentioned as something which support learning and up-skilling	*In the end, I was pushed outside my comfort zone. I had to do my research and really manage the whole job from A to Z. And it was very enriching for me*

For the quantitative data collected through the questionnaire, we calculated the frequency for the IWL strategies (Decius et al., 2019) and for the digital technologies adopted in organised informal learning programmes. For the IWL strategies, we calculated the correlation among the strategies by using the Kendall’s tau test and IBM SPSS Statistics (version 27) predictive analytics software. For the digital technologies adopted in organised informal learning programmes, we calculated the summation of the activities by classifying them into five cognitive engagement categories, based on the ICAP classification integrated with the reflective model by Schon (1983).

## Results

### Disruption

To support the participants in making explicit their informal learning practices, they were initially asked to reflect upon their responses to the disruptions they faced. The topic of disruption was mentioned approximately in the 31% of the total of the units of meaning. The two most mentioned disruptive changes concerned *digital transformation* (O = 153; 41%) and *customers/suppliers* needs (O = 142; 37.9%), followed by *automation* (O = 33; 8.8%), *sustainable development* (O = 32; 8.5%) and *internet of things* (O = 14; 3.8%). Disruptions were often inter-related, as in Extract 1, which shows the co-occurrence between the digital transformation and the customers’ needs.

Extract 1: “*All changes are linked to the change of our customers. We have fewer customers, but they are becoming larger and more professional. Because of digitalisation, our customers need to learn new things*.” (Bioeconomy, manager, Finland)

**Figure 1:**
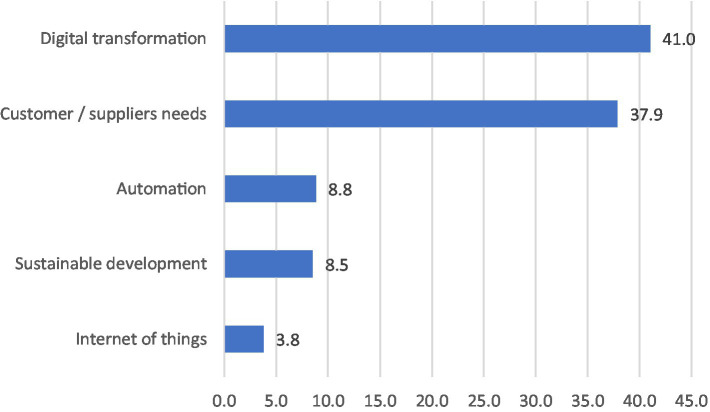
Disruption reported in the total corpus of data. Percentages were calculated through the ratio between the absolute frequency and the total number of units of meaning in which disruptions were mentioned (*N* = 374).

No differences were found among the three countries and the four sectors in terms of perceptions of disruption. The role of the interviewees has a significant effect (*p* < 0.001) only on the perception of the ‘customer and supplier needs’. Indeed, adult educators (O = 13; 5.1%) reported the perception of this disruption less than employers (O = 43; 11.8%) and employees (O = 86; 15.4%). The topic of disruption was mentioned approximately in the 31% of the total of the units of meaning (Figure 1).

The qualitative data showed that *digitalisation* and *customers*/*suppliers’* needs were usually reported as past or present disruptions, whereas *sustainability* and *automation* were reported as imminent and future disruptions. Thus, the highest occurrence of the former two kinds of disruptions compared to the other three could be explained by the concrete challenges that companies have already faced.

For example, in Extract 2, a hotel owner explained that the advent of booking a digital platform (*digitalisation*) was the first kind of disruption he experienced during his career, although years ago.

Extract 2: “*The first big disruptive change in the hotel business was the arrival of* *Booking.com**. This was a turning point, both a blessing and a curse for hoteliers (…) for me, it was the beginning of this period of disruption*.” (Tourism, manager, Switzerland)

However, an employee from the same hotel mentioned the topic of *automation* only at the end of the interview when she was asked to think about future transformations in her sector (Extract 3).

Extract 3: “*People will be replaced more and more, that is, fully automated hotels already exist; it is no longer necessary to have a physical person at the reception… For smaller hotels, automation will require years, due to technologies’ costs of implementation but it will come sooner or later*.” (Tourism, employee, Switzerland)

The awareness of the upcoming disruption was connected to the employees’ need of further organised formal training (Extract 4).

Extract 4: “*In my opinion, the work that I am doing now will become more challenging. With digital transformation and the growing complexity of managing products, I do not think my work would get any easier; on the contrary, it will be more challenging in the future. This work requires, in my opinion, more training in future, because you have to be more aware and to be active in these changings*.” (Bioeconomy, employee, Finland)

### Factors Supporting Learning in Companies That Deal With Disruption

After reflecting on the disruption changes faced, participants were asked to describe which competences they had to develop, how they developed them, and which factors supported their learning in the workplace.

Table 5 presents the result of the co-occurrence between the transversal category ‘support learning’ and the individual and organisational sub-categories, as retrieved by the interview analysis. The strength of the co-occurrence was measured through the Cramer’s V, which indicates medium effects for values higher than 0.3 and large effects for values higher than 0.5 (McHugh, 2013). *Possibilities for social interaction* was the factor with the highest co-occurrence with the category ‘support learning’ (V = 0.325), followed by the individual factor *informal learning strategy* (V = 0.282) and the organisational factor *promotion of autonomy* (V = 0.183).

**Table 5:** Co-occurrence between individual (Ind) and organisational (Org) factors and the sub-category ‘support learning’.

	Absolute frequency	chi	gf	Sign.	V
Org—Reward	20	18.004	1	<0.001	0.124
Org—Connectivity	45	12.040	1	<0.001	0.101
Org—Promotion of autonomy	44	39.611	1	<0.001	0.183
Org—Possibility for social interaction	114	124.537	1	<0.001	0.325
Ind—Attitude toward digital WPL	39	17.566	1	<0.001	0.122
Ind—Motivational factor	66	37.565	1	<0.001	0.178
Ind—Informal learning strategy	97	93.570	1	<0.001	0.282
Ind—Personal learning approach	54	36.114	1	<0.001	0.175
Ind—Occupational self-efficacy	42	19.933	1	<0.001	0.13

**Figure 2:**
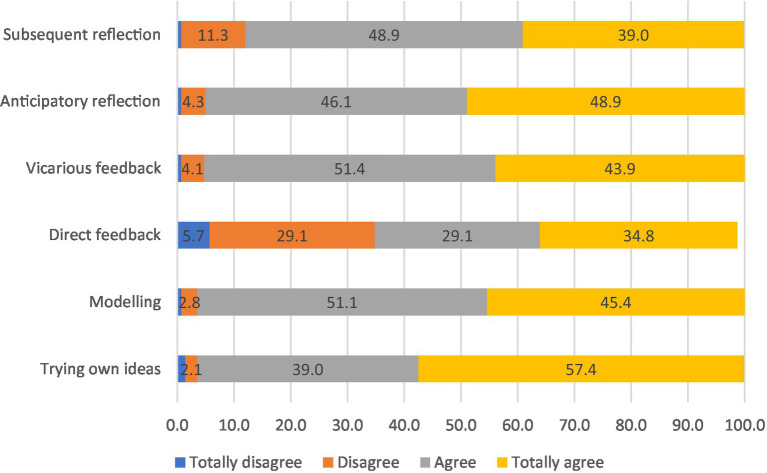
Frequency of informal workplace learning strategies.

The questionnaire allowed us to explore more in-depth the individual IWL strategies that employees of the companies involved in the interviews usually adopt (see Figure 2). *Applying one’s own ideas* (96.4%) and *model learning* (96.6%) were the most frequent IWL strategies adopted by the participants. These two strategies were also retrieved in the interviews. As shown in Extract 5, the IWL strategies of *modelling* and *vicarious feedback* were related to the possibility of having spaces for social interaction with colleagues.

Extract 5: “*Learning happens every day: […] when you do not do your work, but you look what your colleagues are doing […] or the 15-min break, during which people talk about different things: news regarding their duties, industry news, news on destinations or events. It is mainly team building, but you are also learning*.” (Tourism, employee, Italy)

The existence of digital technologies widens opportunities to receive modelling not only from colleagues within the company but also from external actors. For instance, a chef apprentice and an adult trainer in the touristic sector in Switzerland highlighted the role of social media (e.g., Instagram) to provide new forms of modelling on how to execute recipes or to provide services within a restaurant (e.g., how to properly set a table). However, they also highlighted the risks related to identifying the wrong models and the need to be able to critically identify reliable sources for modelling.

*Subsequent reflection* (87.9%) was less reported than *anticipatory reflection* (95%). The lower frequency of subsequent reflection can be explained by the perception of a lack of time to devote to this kind of reflection, as highlighted in Extract 6.

Extract 6: “*Perhaps, companies need to create moments in which we stop and analyse what has happened. I mean, if there is an important project, you have to achieve the objectives; you work to achieve that objective and certainly learn a lot in the process. The problem is that there is almost always a lack of time to analyse what lessons we have learned from this project, what we have done well, what we have done badly, and what we could have done differently. In my opinion, this almost never happens in the company. Instead, it would be invaluable because it would become a company’s know-how, not just something that remains within the individual, which would allow them to improve and improve in all future projects*.” (Textile, employee, Switzerland)

Lastly, *vicarious feedback* (95.3%) was much more reported than *direct feedback* (63.9%). Particularly in the context of Finnish interviews, employees reported the desire to obtain more performance feedback from the supervisor, and more informal feedback from supervisors, colleagues, and customers. Overall, feedback was considered important for continuous learning at work.

We looked for correlations among the six IWL strategies (see Appendix 1). In line with previous research (Decius et al., 2019), the highest correlations were found between the two reflective strategies—*anticipatory reflection* and *subsequent reflection* (tau = 0.600; sign < 0.001)—and the two experience/action strategies—*model learning* and *trying one’s own ideas* (tau = 0.480; sign < 0.001). In contrast to previous research (Decius et al., 2019), the internal correlation between the two feedback strategies, *vicarious* and *direct feedback*, was significant but not very high in the sample (tau = 0.253; sign < 0.001). We found that *vicarious feedback* had a higher correlation with *anticipatory reflection* (tau = 0.338; sign < 0.001) than with *direct feedback* (tau = 0.314; sign < 0.001). On the contrary, *direct feedback* had a higher correlation with *subsequent reflection* (tau = 0.363; sign = 0.001) than with *anticipatory reflection*.

### Approaches Adopted by Companies in Organised Informal Learning and Their Adoption of Digital Technologies

Regarding organised informal learning, companies engage learners mainly in a passive way (O = 92; 7.8%). The remaining four engagement modes were reported quite less frequently: reflective (O = 47; 4%), interactive (O = 19; 1.6%), constructive (O = 17; 1.4%), and active (O = 11; 0.9%). The most frequently reported form of organised informal learning was webinars, probably because of the COVID-pandemic. Other examples of organised informal learning were learning portals, made accessible to all the employees with professional courses and non-formal courses (e.g. yoga and theatre courses). Other organised informal learning practices include more interactive possibilities, such as mobile and video-based learning strategies inspired, to some extent, by social media forms of communication.

Table 6 presents the results of the co-occurrences between the transversal category ‘support learning’ and the five ways in which learners are engaged in organised informal learning programmes. The passive approach has the highest co-occurrence with the transversal sub-category ‘support learning’. However, it is also the only approach significantly associated with the transversal sub-category ‘inhibit learning’, despite the low effect size (chi = 4.403 a; gf = 1; sign = 0.036; V = 0.061). The ambivalence concerning passive-transmissive approaches was mainly reported in relation with webinars. For instance, in Extracts 7 and 8, opposite views emerged concerning the issue of ‘attention’ when attending webinars.

**Table 6:** Co-occurrence of ICAP + R and the sub-category ‘support learning.’

	Absolute frequency	Chi	gf	Sign	V
Active (A)	6	5.678	1	0.017	0.069
Constructive (C)	10	7.918	1	0.005	0.082
Interactive (I)	9	8.676	1	0.003	0.086
Reflective (R)	21	11.473	1	<0.001	0.099
Passive (P)	43	28.276	1	0.000	0.155

Extract 7: “*During these webinars I attended, it was easier for me to take notes. I was much more focused, probably because I was in a less formal place. But I remember much more easily the things that I learnt, I listened to, when I was at home, or in a more comfortable place than in presence at work*.” (Textile, employee, Switzerland)

Extract 8: “*Anything that requires us to relate to each other must be done face-to-face. For the rest, digital is better. Face-to face training cannot be taken away; the human side is required*.” (Tourism, employee, Italy)

**Figure 3:**
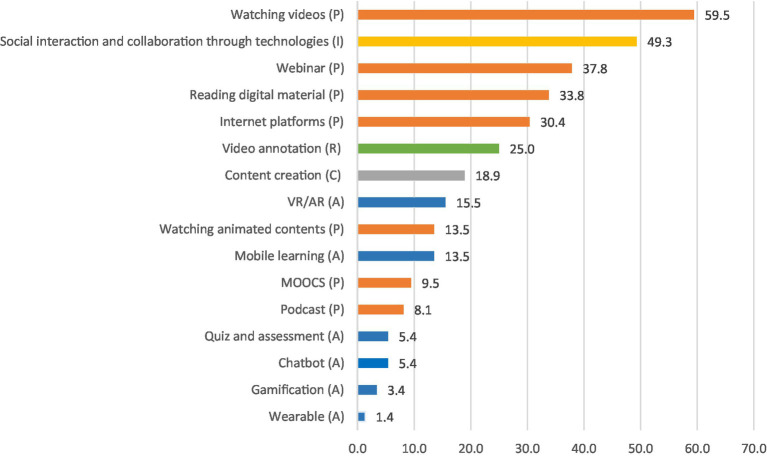
Digital technologies adopted in organised informal learning. Each colour indicates one of the approaches: orange = passive; blue = active; green = reflective; grey = constructive; yellow = interactive.

We used the questionnaire data to collect more detailed information about the use of digital technologies in organised informal training and to understand how digital technologies can be possibly associated with the five cognitive engagement modes. In coherence with the interview results, digital technologies were used frequently in a passive mode, for example, for watching videos (59.5%), attending webinars (37.8%), and reading digital materials (33.8%). Exceptions are ‘social interactions through technologies’ reported by 49.3% of the participants (Figure 3). By aggregating the frequency of the use of technologies in the five cognitive engagement activities, the use of technologies in organised informal learning can be classified as follows: passive 58.3%, interactive 14.9%, active 13.5%, reflective 7.6% and constructive 5.7%.

Although the use of digital technologies was not often associated with reflective activities, a valuable exception was revealed in the interviews (Extract 8).

Extract 8: *We created an internal file where, for each project, we marked everything that we could have done better (…) At the end of the project, we looked at the file and set areas of improvement. Our learning does not remain only in “our memory’ but it is stored in a written analysis. If this had been done not only at the level of the individual team but at the level of the training office in a structured way, I think it would give a very strong push to that process of informal learning I mentioned before.”* (Textile, employee, Switzerland)

## Discussion

The present study explored the informal learning processes activated by companies in response to the perception of past, current and future disruptions in their field. The study’s aim was twofold: first, exploring factors that could support everyday informal learning according to employers, employees, and adult educators employed in companies that deal with disruption. Second, understanding approaches adopted by these companies in organising informal learning.

In line with previous research, the companies involved in our study move across the continuum from tacit, incidental learning to self-regulated and organised informal learning to face disruptive changes (Colley et al., 2003). The results suggest a possible complementarity between the perceived organisational factors that support learning and the reported IWL strategies. *Model learning* and *vicarious feedback* were strongly reported by companies’ representatives in the questionnaire. Complementarily, the interviewees reported that *the possibility of interacting with colleagues* was the main source for supporting everyday informal learning. *Model learning* and *vicarious feedback*, despite being individual strategies, require a social context be exerted. Thus, the high reported frequency of these two IWL strategies could possibly be associated with the high perception of an environment that supports *social interaction*. Similarly, the possibility of frequently *applying one’s own ideas* could be related to an organisational environment that *promotes autonomy*. These results highlight the need to study individual IWL strategies in relation to organisational factors that support learning, or in other words, the organisational learning culture. Previous studies have focused on the relationship between organisational factors and self-regulation strategies (Kittel et al., 2021) and individual learning approaches (Kirby et al., 2003; Froehlich et al., 2015). However, to our knowledge, no studies have focused on IWL strategies as described by Decius et al. (2019) and organisational factors. The Decius model is particularly valuable for studying the relationship between individual and organisational factors, since most of the individual IWL strategies included in the model require a social context to be executed. Thus, in the future, it would be important to understand if there are specific contextual, cultural, and organisational factors that support the emergence of specific IWL strategies.

Interviewees did not report many examples of organised informal learning. Their companies’ learning environment seems to be mainly oriented to everyday, tacit, and self-regulated informal learning. During organised informal learning, employees were mainly engaged in passive ways, for example, through webinars. Interviewees recognised the shortcomings of transmissive online training; they reported the lack of a social dimension and a separation from everyday work, especially in comparison to face-to-face training organised at work. Despite the acknowledged limitations, they did not present many alternative examples of the use of digital technologies in organised informal learning. Most of the participants, including managers and adult educators, did not show the awareness that digital technologies can be exploited to actively engage people, for example, to support reflection on the experience or to construct individually or collaboratively knowledge artefacts (Nonaka and Konno, 1998; Schwendimann et al., 2015). It seems that participants believed that higher forms of cognitive engagement are possible only within face-to-face organised informal training or in everyday informal learning. However, there are some forms of cognitive engagement that are rarely incidental and spontaneous, such as transforming tacit knowledge into explicit knowledge through reflective activities (Kolb, 1984; Clark et al., 2018; Cattaneo and Motta, 2021). Although the companies involved in this study scarcely mentioned reflective activities in organised informal learning programmes, participants frequently reported *anticipatory reflection* as an IWL strategy. The unexpected high frequency of *anticipatory reflection* could be explained by the disruptive nature of the workplace in which people are involved. Interviewees with different roles and expertise seemed to be quite aware of the disruptions their workplaces were facing. This awareness was probably brought about by the concrete implications that disruption is having on their daily work. Navigating in a changing context forced employees to reflect on how to carry out new tasks. However, *subsequent reflection* was less reported than *anticipatory reflection*. Subsequent reflection, as suggested by an employee (Extract 6), needs to be structured (Kramarski and Kohen, 2017), especially in disruptive contexts where people feel the pressure to adapt to rapidly changing contexts.

In line with previous research, we found a good internal correlation between the two reflective strategies of anticipatory reflection and subsequent reflection and the two experience/action strategies. By contrast, the internal correlation between the two feedback strategies—vicarious and direct feedback—was not high as expected (Decius et al., 2019). We found that vicarious feedback correlated more with anticipatory reflection. Conversely, direct feedback correlated more with subsequent reflection. These results could be interpreted again as the central role of self-regulated learning strategies required in disruptive contexts. *Vicarious feedback* and *modelling* were probably IWL strategies adopted by the employees to collect materials on which to reflect before executing a new task. However, *direct feedback* was less likely to occur spontaneously and needed to be structured in *organised informal learning programmes* to support *subsequent reflection*.

In summary, the companies facing disruption involved in this study seem to mainly rely on everyday informal learning and self-regulated learning. For this company, the social dimension seems to be the main source of support for learning, a space where people can meet face-to-face and share feelings, experiences, and mental models. Disruptive changes require employees to self-regulate their learning by reflecting before performing a new task. Also, in this case, the social space allows us to reduce the ambiguity of the new task, because people learn looking for models and peers’ experience. Multiple contributing factors can explain the focus on knowledge transfer in organised informal learning. First, the pandemic requires that organised informal learning be carried out through the adoption of digital technologies. Second, companies showed a low level of awareness concerning how digital technologies can be used for active learning, reflective, and meta-cognitive learning, and constructive and interactive learning. Third, the time pressure connected to the disruption does not provide much space for supporting subsequent reflection through direct feedback activities.

This study has some limitations. First, we involved a small and heterogeneous group of participants in both the interviews and the questionnaire. Based on the availability provided by the companies, it was not possible to involve companies from the same fields in the three countries, which would have allowed for a more structured comparison within similar productive sectors. Second, since the questionnaire was anonymous, we were unable to triangulate data for each interviewee. It was not possible to test whether the association between the perceived organisational factors and individual IWL strategies is significant from a statistical point of view since data were collected through different sources. Another limitation is that we did not have detailed information concerning the use of digital technologies in organised informal learning programmes. Thus, the attribution of the different uses of technologies to the five cognitive engagement modes (ICAP + R) could in some cases be partial. The study would have benefited from a longitudinal research design for tracking and detecting ongoing changes and transitions in skills and competences.

Despite the abovementioned limitations, this study provides insight into the factors supporting informal learning in companies which are facing disruptions. Based on our results, it is possible to provide some indications for future research and practices. Future research could better explore, from an empirical perspective, the complex relationship between perceived organisational learning factors and informal workplace learning strategies by involving larger samples within different kinds of companies. In the future, it would also be interesting to develop a model to describe the technology integration of digital technologies in organised informal workplace programmes. Our results suggest that integrating the ICAP model (Chi et al., 2018) with the reflective theory (Schon, 1983) could be a good starting point. Based on this model, a scale could be developed and validated (see, e.g. Sailer et al., 2021) to study how to improve digital technology integration in their organised informal learning programmes. This scale can be used by companies as a self-assess tool to understand how to improve their use of digital technologies to support informal workplace learning. Moreover, in the future, it would be interesting to carry out action research in companies facing disruption through the theoretical lens of knowledge creation (Nonaka and Konno, 1998; Schwendimann et al., 2015). These theories are particularly interesting because they provide guidelines regarding how to shift from a social dimension of learning to externalisation through reflection and reification.

From a practical perspective, companies’ trainers and HR services could benefit from training concerning the importance of organised informal learning. Although incidental and self-regulated learning are important sources of learning (Marsick et al., 2006, 2008), contemporary work requires that employees acquire appropriate conceptual and symbolic knowledge that is not immediately evident or accessible in workplaces (Billett, 2018). Knowledge, tools, and working methods develop rapidly, especially in contexts that deal with disruption. Therefore, incidental workplace learning is not always enough, and more formal and intentional learning, as well as guidance and evaluation, is needed (Clark et al., 2018). Organised informal learning could be enhanced by digital technologies, supporting advanced forms of cognitive engagement, for example, by providing direct feedback in the form of meta-cognitive prompts to support reflection (Cattaneo and Motta, 2021) or integrating subsequent reflection with modelling activities (Etscheidt et al., 2012).

In the future, we will need to carry out interventions and research to support companies in adopting digital technologies to bridge formal and informal workplace learning, tacit and explicit knowledge and action with reflection.

## Data Availability Statement

The raw data supporting the conclusions of this article will be made available by the authors, without undue reservation.

## Ethics Statement

The studies involving human participants were reviewed and approved by Häme University of Applied Sciences (HAMK). The patients/participants provided their written informed consent to participate in this study.

## Author Contributions

FA, ER, and AC contributed to the conception and design of the study. FA organized the database, performed the statistical analysis, and wrote the first draft of the manuscript. ER and KM carried out the qualitative data collection and data analysis in the Finnish context and wrote sections of the manuscript. All authors contributed to manuscript revision, read, and approved the submitted version.

## Funding

The research project has been funded with support from the European Commission, under ERASMUS+, with the reference number 2020-1-FI01-KA204-066655.

## Conflict of Interest

The authors declare that the research was conducted in the absence of any commercial or financial relationships that could be construed as a potential conflict of interest.

## Publisher’s Note

All claims expressed in this article are solely those of the authors and do not necessarily represent those of their affiliated organizations, or those of the publisher, the editors and the reviewers. Any product that may be evaluated in this article, or claim that may be made by its manufacturer, is not guaranteed or endorsed by the publisher.

## Appendix 1

Internal correlation among the six IWL strategies.

**Table:**

	Trying own ideas	Model learning	Direct feedback	Vicarious feedback	Subsequent reflection	Anticipatory reflection
*Trying own ideas*						
Kendall’s tau	1	0.480^**^	0.280^**^	0.193^*^	0.278^**^	0.403^**^
Sig.	.	<0.001	<0.001	0.018	<0.001	<0.001
*Model learning*						
Kendall’s tau	0.480^**^	1	0.374^**^	0.460^**^	0.301^**^	0.418^**^
Sig.	<0.001	.	<0.001	<0.001	<0.001	<0.001
*Direct feedback*						
Kendall’s tau	0.280^**^	0.374^**^	1	0.253^**^	0.363	0.314
Sig.	<0.001	<0.001	.	0.001	<0.001	<0.001
*Vicarious feedback*						
Kendall’s tau	0.193^*^	0.460^**^	0.253^**^	1	0.288^**^	0.338^**^
Sig.	0.018	<0.001	0.001	.	<0.001	<0.001
*Subsequent reflection*						
Kendall’s tau	0.278^**^	0.301^**^	0.363^**^	0.288^**^	1	0.600**
Sig.	<0.001	<0.001	<0.001	<0.001	.	<0.001
*Anticipatory reflection*						
Kendall’s tau	0.403^**^	0.418^**^	0.314^**^	0.338^**^	0.600^**^	1
Sig.	<0.001	<0.001	<0.001	<0.001	<0.001	.

*Indicates *p* < 0.05. **Indicates *p* < 0.01.
